# The HER4-YAP1 axis promotes trastuzumab resistance in HER2-positive gastric cancer by inducing epithelial and mesenchymal transition

**DOI:** 10.1038/s41388-018-0204-5

**Published:** 2018-03-14

**Authors:** Jiaolong Shi, Fengping Li, Xingxing Yao, Tingyu Mou, Zhijun Xu, Zheng Han, Siyu Chen, Wende Li, Jiang Yu, Xiaolong Qi, Hao Liu, Guoxin Li

**Affiliations:** 1 Department of General Surgery, Nanfang Hospital, Southern Medical University, Guangdong Provincial Engineering Technology Research Center of Minimally Invasive Surgery, Guangzhou, China; 2 Department of Guangdong Laboratory Animals Monitoring Institute, Guangdong Key Laboratory of Laboratory Animals, Guangzhou, China

## Abstract

Trastuzumab is the only target to be approved as the first-line treatment of HER2 positive metastatic gastric cancer, but ubiquitous resistance decreases its therapeutic benefit. In this study, we found HER4, phosphorylation HER4 (p-HER4) and the mesenchymal marker Vimentin increased in trastuzumab-resistant cells (MKN45TR and NCI-N87TR), while epithelial markers expressions in trastuzumab-resistant cell lines and animal models decreased. Additionally, silencing HER4 prevented the epithelial-mesenchymal transition and led to decreased proliferation and migration in vitro and in vivo. The expression of YAP1, a vital downstream interacted target of HER4, decreased when HER4 was knocked down. Interestingly, stimulation of NRG1 could compromise the inhibitory impact and rescue cell survival; whereas, transfection of siYAP1 sensitized trastuzumab-treated cells. Expression analysis of the proteins in patient-derived xenograft model (PDX) mice showed that HER4, p-HER4, YAP1, and Vimentin were clearly upregulated in the trastuzumab-resistant mice compared to mice without trastuzumab resistance. However, HER2 and E-cadherin were downregulated in response to continuous treatment with trastuzumab. These findings elucidated that the central role of the HER4-YAP1 axis in trastuzumab resistance of HER2-positive gastric cancer cells through induction of EMT. Hence, regulating the HER4-YAP1 axis might be a promising strategy for clinical interventions in patients with HER2-positive gastric cancer.

## Introduction

Gastric cancer is the fifth most commonly diagnosed malignancy and the third leading cause of cancer death worldwide [1]. Adjuvant chemotherapy following radical surgical resection would specifically benefit patients with advanced gastric cancer [2], but the high heterogeneity of the disease leads to metastasis and recurrence, with a significantly poorer prognosis that is <1-year median survival and a 5-year survival rate <7% [3, 4]. Among the various genomic events, abnormal expression of HER2, a prognostic factor for patients, is involved in as many as 7–34% of gastric cancers [5–7]. HER2 acts as a co-receptor that modulates signals after ligands bind to other receptors in the epithelial growth factor receptor (EGFR) family. By interacting independently with extracellular ligands or by heterodimerizing with other members of the ErbB family, downstream signaling pathways, including the MAPK and PI3K/AKT/mTOR pathways, are activated to facilitate uncontrolled cell growth and tumorigenesis [8]. Trastuzumab, the only target agent approved by the FDA as a first-line treatment of metastatic gastric adenocarcinoma, substantially improves the outcome for patients with HER2-positive gastric cancer [9, 10]. However, most patients become refractory to the trastuzumab-based treatment within 1 year [11]. Although new agents have been explored to delay the emergence of resistance, acquired resistance still limits the duration of the response to trastuzumab [12]. Thus, a characterization of the resistance mechanism of trastuzumab is urgently needed to offer an alternative option for patients who would suffer from inevitable resistance.

Currently, a large proportion of investigations about the molecular mechanisms of trastuzumab resistance stem from breast cancer, and the generally recognized processes include hyperactivation of the phosphatidylinositol-3-kinase (PI3K) pathway by PI3K alterations or PTEN loss [13–15]. Scaltriti et al.[16] demonstrated that p95HER2, a mutated form of HER2, did not bind to trastuzumab and retained its tyrosine kinase activity, which partly accounted for trastuzumab resistance. Recent studies also uncovered that growth factors act as ligands of receptor tyrosine kinases, as well as upregulation of other growth factors, such as NRG1 and HGF, which also bind to HER2, can confer resistance to anti-HER2 drugs [17, 18]. Piro et al.[11] found that expression of fibroblast growth factor receptor 3 (FGFR3) could stimulate epithelial-to-mesenchymal transition (EMT) after resistance developed in gastric cancer, with the FGFR3/AKT axis as the presumed escape pathway responsible for trastuzumab resistance. However, the exact mechanisms of trastuzumab resistance in HER2-positive gastric cancer still remained unknown.

As a member of the ErbB family, HER3 can dimerize with HER2, transcriptional and posttranslational upregulation of HER3 were suggested to promote the phosphorylation of a residue on HER2, thus maintaining activation of the PI3K pathway; an anti-HER3 agent in combination with inhibitors of HER2 and the PI3K pathway were recommended to achieve effective treatment [19]. In HER2-positive gastric cancer, dual inhibition of EGFR and HER2 displayed a satisfactory killing ability toward trastuzumab-resistant cells [20, 21]. HER4, another member of the ErbB family, also reportedly impacts HER2-positive cancer cell survival after cells become resistant to trastuzumab, and nuclear localization of HER2 indicateed a poor prognosis in breast cancer [22, 23]. The presence of HER4 sensitizes HER2-positive cells to trastuzumab and was considered a potential target for overcoming trastuzumab resistance [24]. But the exactly role and mechanism of HER4 in HER2 positive gastric cancer trastuzumab resistance still remains unknown. In gastric cancer, NRG1 associates with its receptors, HER3 and HER4, might be an independent and unfavorable prognostic factor and a therapeutic target.

In previous study, we discovered that a somatic mutation in HER4 was associated with peritoneal metastasis of gastric adenocarcinoma [25]. The results of this study determined its role in EMT-induced trastuzumab resistance by regulating vital downstream target YAP1 and activating PI3K signal pathway. Importantly, we successfully established HER2 positive PDX model and depend our understanding on the important role of HER4-YAP1-EMT axis in inducing trastuzumab resistance. Thus, further investigation was conducted to clarify the exact mechanism by which HER4 regulates the acquisition of trastuzumab resistance.

## Results

### HER2 positive gastric cancer cells promote EMT after becoming resistant to trastuzumab

**Fig 1:**
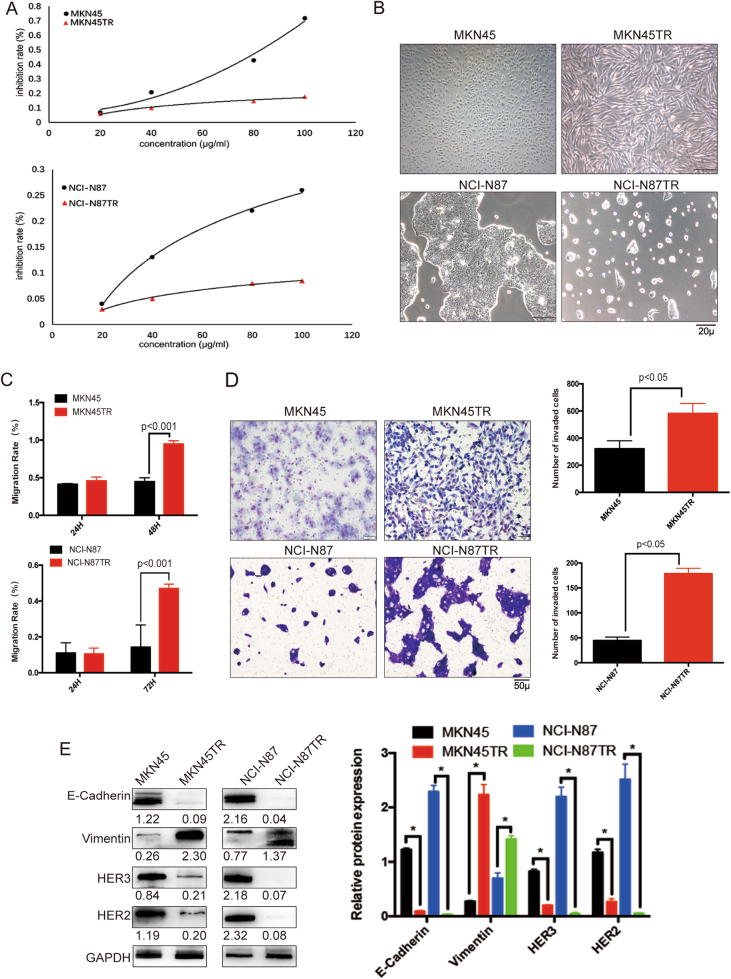
HER2 positive gastric cancer cells promote EMT after becoming resistant to trastuzumab. **a** Establishment of the MKN45TR and NCI-N87TR cell lines and resistant curve determined by inhibition rate. **b** The morphology of the MKN45TR and NCI-N87TR cells and parental MKN45 and NCI-N87 cells was observed by using phase-contrast microscopy. **c** Data from the wound healing analysis of the MKN45, NCI-N87 and MKN45TR, NCI-N87TR cells. **d** Representative figures and data from the transwell assay of the MKN45, MKN45TR cells for 24 h and NCI-N87, NCI-N87TR cells for 72 h. Each bar represented the mean ± SD. The results were reproduced in three independent experiments. **e** Western blot analysis for the expression of ErbB family receptors and EMT-associated markers in MKN45TR and NCI-N87TR cells. The data are presented as mean ± SD of three independent experiments

**Fig. 2:**
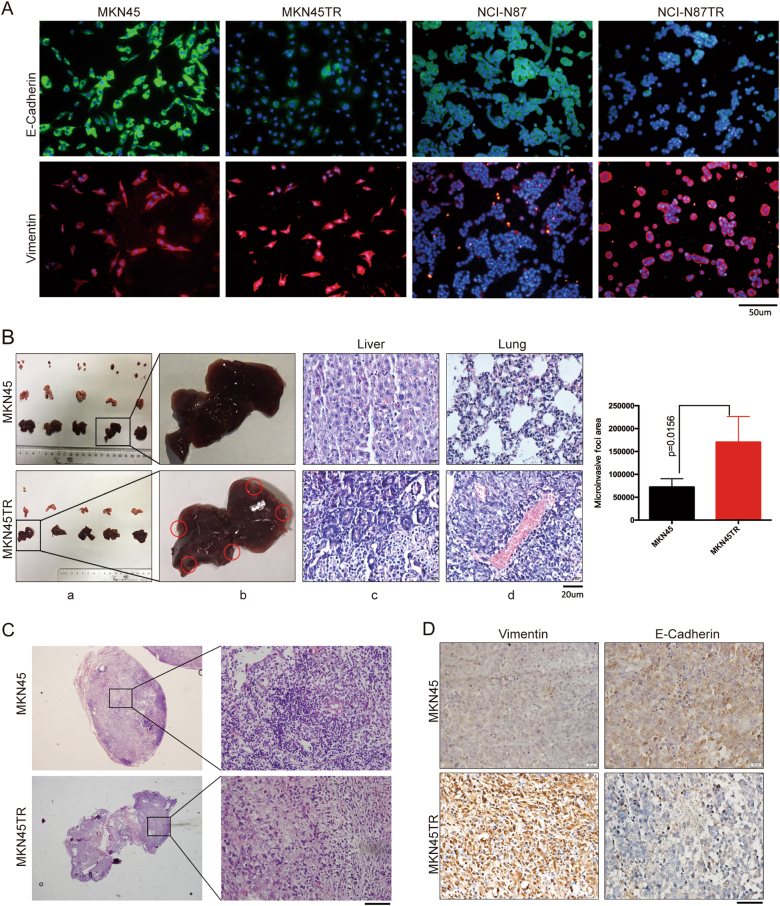
Trastuzumab-resistant cells are highly aggressive in vivo. **a** Subcellular localizations of EMT-associated markers E-Cadherin and Vimentin in MKN45, NCI-N87, and MKN45TR, NCI-N87TR cells was assessed through immunofluorescence. **b** 1 × 10^5^ MKN45 and MKN45TR cells were injected into nude mice through the tail vein for 30 days to evaluate the lungs, livers, and subcutaneous homing potential. Representative figures for liver metastatic tumor (**b**), HE staining for metastatic liver and lung (**c**, **d**). Data for the lung micro invasive foci area, the area of metastatic lung nodules in individual mice was calculated. **c** HE staining for subcutaneous homing of MKN45 and MKN45TR cells by tail vein injection. **d** IHC analysis of the expression change of Vimentin and E-Cadherin between MKN45 and MKN45TR group. Representative figures are shown. The data are presented as mean ± SD of three independent experiments

It is possible that multiple resistance mechanisms may coexist in patients with HER2 positive metastatic gastric cancer [26]. To identify and target the key nodes of trastuzumab resistance, we generated trastuzumab-resistant gastric cancer cell lines using HER2-positive MKN45 and NCI-N87 cells. Cells were exposed to increasing concentrations of trastuzumab for 8 months, and single-cell clones were isolated from a pool of resistant cells to generate the resultant cell lines. Compared with parental cells, MKN45TR (MKN45 trastuzumab-resistant cells) and NCI-N87TR (NCI-N87 trastuzumab-resistant cells) exhibited significantly higher resistance to the trastuzumab treatment in vitro (MKN45TR, IC50 = 99,250.70 µg/ml, RI = 31.33, *p* < 0.01, NCI-N87TR, IC50 = 620,570.69 µg/ml, RI = 1032.95, *p* < 0.01), and the resistance curve was different (Fig. 1a). Furthermore, trastuzumab cells displayed a long spindle-shaped morphology with thin, long pseudopods, which occasionally resembled finger-like pseudopods that extended from the cell bodies (Fig. 1b). Wound healing and Transwell assays showed that migration and invasive capacities of resistant cells were higher than parental cells (Fig. 1c, d and Supplementary Figure 1A). These findings demonstrated that activation of EMT occurred after parental cells became resistant to trastuzumab. To further dissect this observation, EMT markers were measured using western blot and immunofluorescence. The results revealed that levels of mesenchymal markers Vimentin increased while epithelial markers E-Cadherin decreased. Additionally, Western blot analyses also showed significant expression decrease of other two ErbB family member HER2 and HER3 in trastuzumab-resistant lines. (Figs. 1e and 2a).

### Trastuzumab resistance in MKN45 cells promote metastasis in vivo

Since the investigation revealed that migration and invasion ability was elevated upon trastuzumab resistance acquired, the homing capacity of cancer cells in vivo was further investigated. We performed tail vein injections of cancer cells in nude mice to determine the rate of nodule formation in the lungs and livers. Compared with MKN45 group, larger tumor nodules were observed in the lungs and livers in MKN45TR group. (Fig. 2b). Interestingly, we found multiple subcutaneous nodules in both groups. The HE staining analysis confirmed that the subcutaneous nodules in MKN45 group were all inflammatory cell infiltrations while that in MKN45TR group were all metastatic cancer cells (Fig. 2c). Once resistance acquired, E-Cadherin expression was decreased, and Vimentin was increased in vivo (Fig. 2d). These observations indicated that during resistance process, the homing capacity became increasingly stronger than parental cells.

### HER4 promotes trastuzumab resistance by inducing EMT

Previous research revealed that EMT was involved in trastuzumab resistance in HER2 positive gastric cancer. To gain insight into possible mechanism of the observed phenotypic transition, we examined our previous whole-exome sequencing results, with emphasis on the potential role HER4 played in inducing gastric cancer cell EMT and metastasis (Supplementary Figure 1B).

**Fig. 3:**
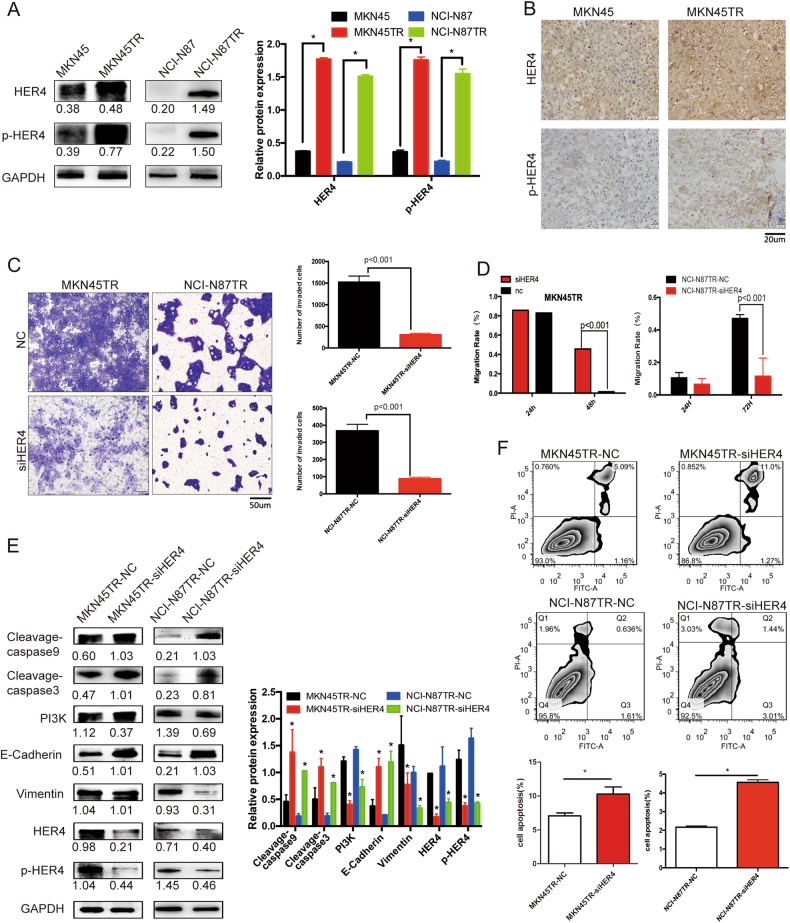
HER4 promotes trastuzumab resistance by inducing EMT. **a** HER4 and p-HER4 protein contents in MKN45, NCI-N87, MKN45TR, and NCI-N87TR cells were performed by western blotting analysis. **b** IHC analysis expression of HER4 and p-HER4 in MKN45TR subcutaneous tumor compared with MKN45 group. **c** Transwell analysis of MKN45TR and NCI-N87TR cells transfected with NC or siHER4 for 24 h. Five randomly selected fields were assessed under a microscope. The bars in the lower panel represent the number of invaded cells. **d** The wound healing assay was performed to assess the migration capacity of HER4 in MKN45TR cells for 24 h and NCI-N87TR cells for 72 h. **e** Western blot analysis of HER4, p-HER4, Vimentin, E-Cadherin, PI3K, and apoptosis associated protein Cleavage-caspase3 and Cleavage-caspase9 in MKN45TR and NCI-N87TR transfected with NC or siHER4 for 48 h cells. **f** MKN45TR and NCI-N87TR cells were treated with siRNA or NC for 24 h. Apoptotic rates were measured by flow cytometric analysis using Annexin V staining. The data are presented as mean ± SD of three independent experiments

HER4, as a member of the ErbB family, could impact on HER2-positive cancer cell survival after cells become resistant to trastuzumab [22, 23]. As indicated by western blot analyses, HER4 and p-HER4 expressions in resistant cells were increased compared to the parental cells (Fig. 3a). IHC showed that HER4 and p-HER4 were overexpressed in the MKN45TR subcutaneous tumor compared with MKN45 group (Fig. 3b). To confirm the necessity of HER4 in EMT process of trastuzumab resistance, we selected an efficient HER4-targeted siRNA fragment by western blot and Q-PCR (Supplementary Figure 2A). Transwell and wound healing assays showed that invasive and migration capacities of the resistant-siHER4 cells were lower than that of resistant-NC cells (Fig. 3c, d and Supplementary Figure 2B). After the HER4 knockdown, Vimentin expression decreased while E-Cadherin increased. Meanwhile, the resistance-associated molecular pathway, PI3K pathway, was inactivated after siHER4 silencing. Besides, overexpression of Cleavage-caspase3 and Cleavage-caspase9 indicated that siHER4 could promote resistant cells apoptosis (Fig. 3e). By using Annexin V staining, flow cytometric analysis showed increased percentage of cells undergoing apoptosis after the siHER4 transfection compared to the NC (*p* < 0.05, Fig. 3f).

**Fig. 4:**
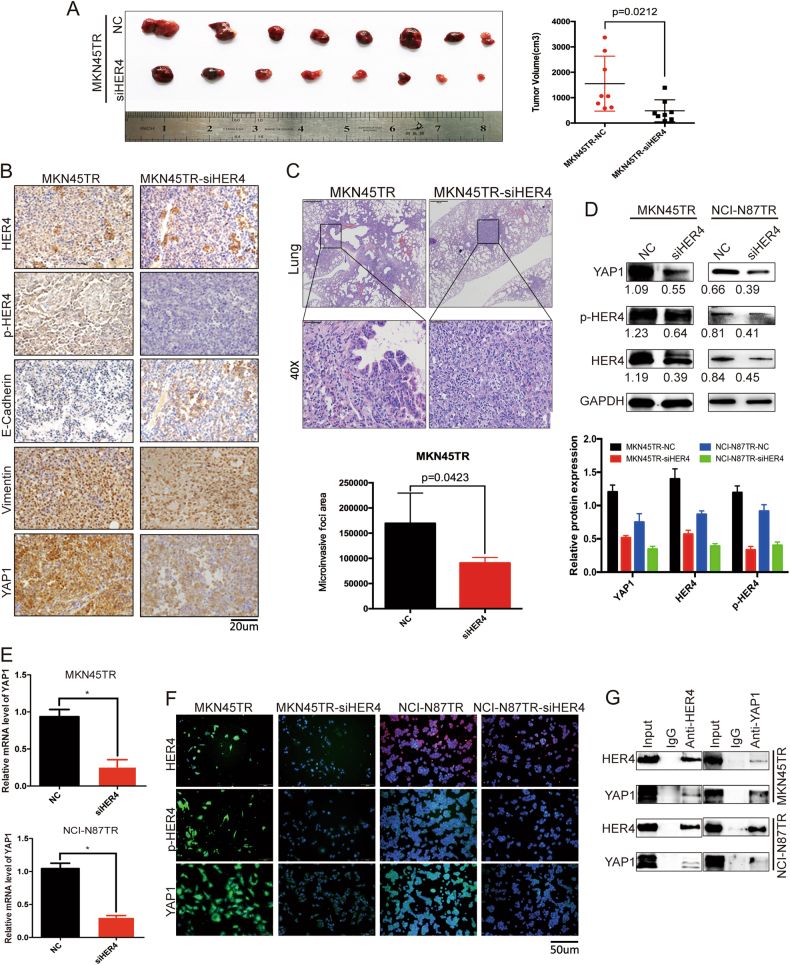
HER4 promotes trastuzumab resistance in vivo and inducing EMT by regulating YAP1. **a** Tumor samples of the two groups were collected 30 days after 2 × 10^6^ MKN45TR-NC and MKN45TR-siHER4 cells subcutaneous implantations when tumor sizes reached the sacrificed endpoints. The sizes of the tumors were measured in triplicated. **b** Representative figures from the subcutaneous tumor IHC analysis of HER4, p-HER4, YAP1, E-Cadherin, and Vimentin are shown. **c** 1 × 10^5^ MKN45TR-NC or MKN45TR-siHER4 cells were injected by tail vein for 30 days and the magnified areas indicate the areas with metastatic nodes by HE staining. **d** Western blot analysis of the potential downstream target YAP1 of HER4 for MKN45TR and NCI-N87TR cells with or without transfection of siHER4. **e** Q-PCR analysis of relative YAP1 mRNA levels in MKN45TR and NCI-N87TR cells transfected with NC or siHER4 for 24 h. **f** Immunofluorescence of YAP1 expression after the siHER4 was transfected into MKN45TR or NCI-N87TR cells for 24 h. **g** The interaction between HER4 and YAP1 was measured by co-immunoprecipitation analysis

The lentivirus-transfected cell lines with stably knocked-down expression of HER4 were established and the fluorescence showed transfected efficiency (Supplementary Figure 2C). As shown in Fig. 4a, b, growth rate was decreased, and Vimentin expression was reduced while E-Cadherin was increased in tumors of siHER4 group compared with NC group. Besides, HER4 stably knocked-down MKN45TR cells were employed to investigate homing capacity of cancer cells in nude mice. Compared to the animal models of NC group, smaller tumor nodules were found in the lungs of the siHER4 group (*p* = 0.0423, Fig. 4c). These results further confirmed that HER4 played an important role in promoting trasutuzumab resistance by regulating EMT.

### The HER4-YAP1 axis, through its induction of EMT, is essential for trastuzumab resistance

**Fig. 5:**
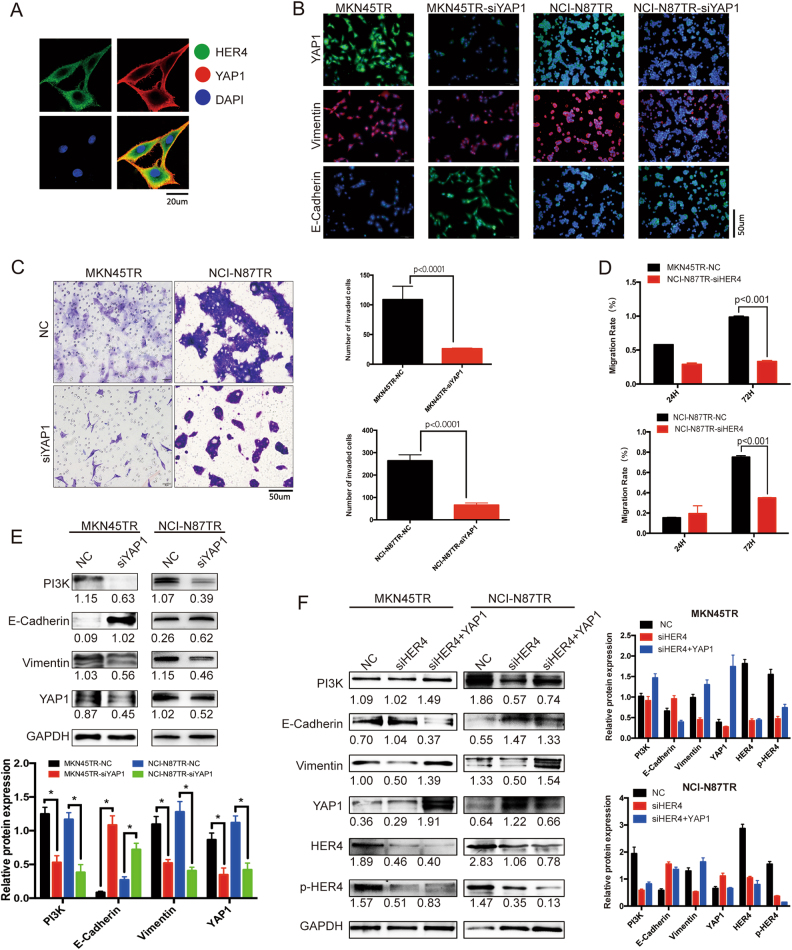
The HER4-YAP1 axis, through its induction of EMT, is essential for trastuzumab resistance. **a** Confocal microscopy was performed to investigate the subcellular co-localization of HER4 and downstream YAP1. Merged images in which the expression of HER4 is showed in green and YAP1 expression is shown in red. The yellow fluorescence represents the co-expression of HER4 and YAP1. Nuclear is counterstained with DAPI (blue). **b** Subcellular localizations of YAP1, E-Cadherin, and Vimentin in siYAP1-transfected MKN45TR and NCI-N87TR cells, as analyzed with immunofluorescence. **c** Transwell was conducted to measure the invasion capability of MKN45TR and NCI-N87TR cells after transfected with NC or siYAP1 for 48 h. **d** Wound healing assays was used to measure the invasion capability of MKN45TR and NCI-N87TR cells after transfected with NC or siYAP1 for 72 h. **e** Western blot analyses of the relative expression levels of EMT-associated markers and the PI3K signaling pathway after NC or siYAP1 was transfected for 48 h. The data are presented as mean ± SD of three independent experiments. **f** MKN45TR and NCI-N87TR cells were simultaneously coinfected into siHER4 and YAP1 cDNA for 48 h. The expression of PI3K, E-Cadherin, Vimentin, YAP1, HER4, and p-HER4 were detected by western blot. (color figure online)

To investigate potential role of HER4 in promoting resistant cells EMT, it is predicted that YAP1 might be an important downstream molecule as an EMT inducer by using String.com network (Supplementary Figure 3A). Previous IHC and western blot, Q-PCR, immunofluorescence results revealed that YAP1 expression decreased when HER4 was knocked down in vitro and in vivo (Fig. 4b, d–f). Additionally, immunoprecipitation analysis of resistant cells showed mutual interaction between HER4 and YAP1 (Fig. 4g). Then, confocal microscopy analysis revealed that HER4 and YAP1 co-localized to the cytosol and cytoplasmic membrane in MKN45TR cells (Fig. 5a). Those data indicated that YAP1, interacted with HER4, might play an important role in downstream of HER4. As Immunofluorescence shown, the level of Vimentin decreased while E-Cadherin increased when YAP1 was knocked down (Fig. 5b). Transwell and wound healing assays also revealed lower invasive and migration capacity of siYAP1 cells compared to NC group (Fig. 5c, d and Supplementary Figure 3B). Western blot analysis confirmed reduction of Vimentin in response to YAP1 silencing in resistant cells. Meanwhile, suppressed YAP1 lead to inhibition of PI3K signaling pathway (Fig. 5e). Then, resistant cells were simultaneously coinfected into siHER4 and YAP1 cDNA to explore the role of YAP1 in HER4-mediated EMT and trastuzumab resistance. The effects of siHER4 on inhibiting resistant cells EMT and PI3K-signal pathway were significantly improved (Fig. 5f). It was hence proposed that YAP1 might be a demonstrably downstream effector of HER4 and through which HER4 exerted its EMT effects on trastuzumab-resistant cells.

As shown in Supplementary Figure 3C and D, the MKN45TR morphology became epithelial in shape and that MKN45TR cells exhibited attenuated levels of p-HER4, HER4, Vimentin and PI3K along with increased levels of epithelial proteins after trastuzumab was withdrawn. These observations indicated that maintenance of EMT required continuous stimulation of trastuzumab. Taken together, those data revealed that HER4-YAP1 axis, through its induction of EMT, might be crucial in process of trastuzumab resistance.

### NRG1, through its activation of the HER4-YAP1 axis, is essential for EMT-induced trastuzumab resistance

**Fig. 6:**
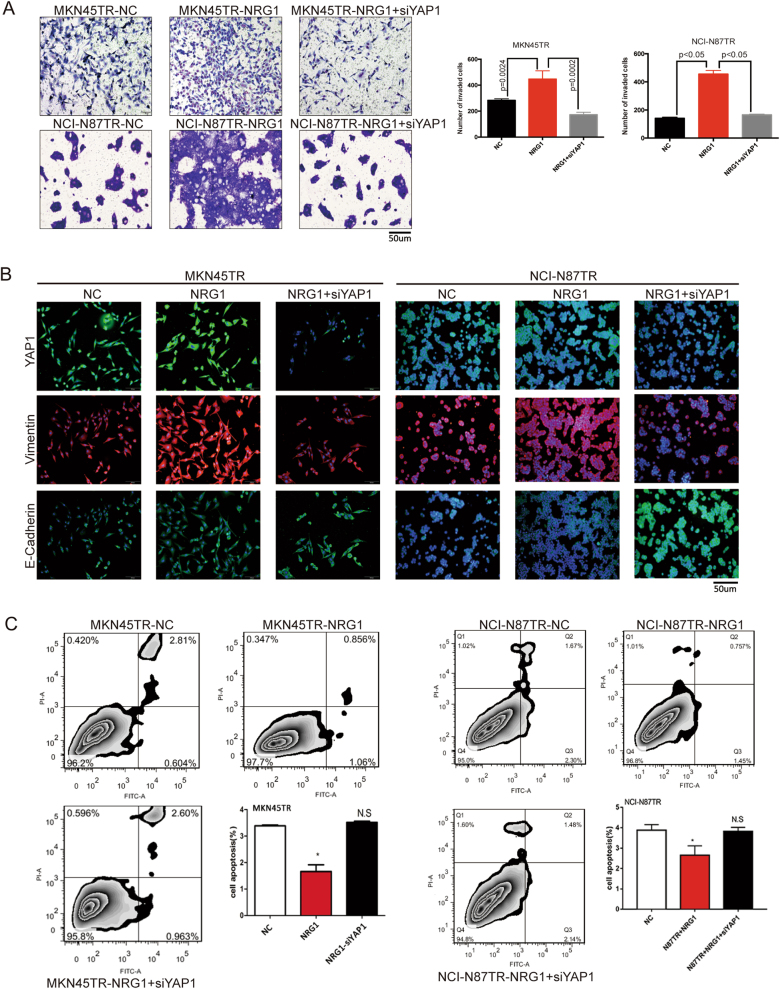
NRG1, through its activation of the HER4-YAP1 axis, is essential for EMT-induced trastuzumab resistance. **a** MKN45TR or NCI-N87TR cells were treated with or without siYAP1 for 24 h and then incubated with or without 100 ng/ml NRG1 for 20 or 30 min, respectively. And the invasion ability was measured by Transwell assay. **b** MKN45TR or NCI-N87TR cells were treated with or without siYAP1 for 24 h and then incubated with or without 100 ng/ml NRG1 for 20 or 30 min, respectively. And then, immunofluorescence was employed to detect the expression of Vimentin and E-Cadherin. **c** MKN45TR or NCI-N87TR cells were treated with or without siYAP1 for 24 h and then incubated with or without 100 ng/ml NRG1 for 20 or 30 min, respectively. The flow cytometric was used to detect the effect on MKN45TR and NCI-N87TR cell apoptosis after NRG1 stimulation and YAP1 knockdown by Annexin V staining. The data are presented as mean ± SD of three independent experiments

**Fig. 7:**
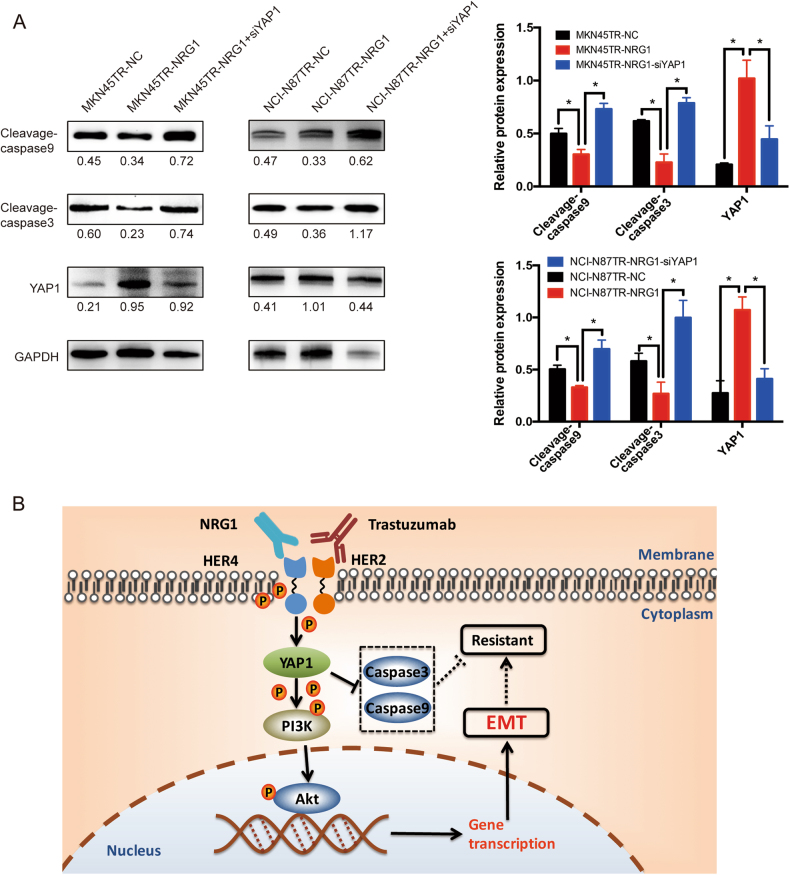
Diagram summarizing the role of HER4 in trastuzumab resistance regulation in HER2 positive gastric cancer. **a** MKN45TR or NCI-N87TR cells were treated with or without siYAP1 for 48 h and then incubated with or without 100 ng/ml NRG1 for 20 or 30 min, respectively. Western blot analysis was conducted to detect the expression of Cleavage-caspase3 and Cleavage-caspase9. **b** Hypothetic model illustrating that HER4-YAP1 axis induced EMT by activating PI3K signal pathway to promote trastuzumab resistance. Meanwhile, HER4-YAP1 also inhabit apoptosis associated protein Cleavage-caspase3 and Cleavage-caspase9, which has closely relationship with trastuzumab resistance

To determine whether stimulation of full-length ErbB4 by its ligand, NRG1, was sufficient to induce YAP1 expression and EMT, resistant cells were stimulated with 100 ng/ml NRG1 at different time points and optimized the stimulation time to 20 min in MKN45TR cells and 30 min in NCI-N87TR cells, respectively (Supplementary Figure 4A). The results of Transwell assays indicated that NRG1 promoted invasion of resistant cells and that YAP1 silencing reduced activity of NRG1 (Fig. 6a). The immunofluorescence assay revealed that NRG1 upregulated Vimentin and downregulated E-Cadherin, whereas YAP1 silencing reversed the activity (Fig. 6b). Additionally, NRG1 stimulation reduced the apoptosis rate of resistant cells, and YAP1 silencing offset suppression of apoptosis (*p* < 0.05, Fig. 6c). Western blot showed that NRG1 stimulation could reduce expressions of apoptotic protein Cleavage-caspase3 and Cleavage-caspase9 while YAP1 silencing could rescue its expression (Fig. 7a). Those results indicated that NRG1 was essential for EMT-induced trastuzumab resistance through the activation of the HER4-YAP1 axis.

Based on the above, these data revealed that HER4-YAP1 axis might regulate acquired resistance to trastuzumab in HER2-positive gastric cancer cells by promoting EMT. The axis conferred an increased proliferation capacity of HER2-positive gastric cancer cells exposed to trastuzumab. The abnormal expressions of HER4 and YAP1 increased activation of PI3K to promote resistance to the trastuzumab treatment (Fig. 7b).

### The HER4-YAP1 axis is associated with trastuzumab resistance in a HER2-positive patient-derived xenograft model

**Fig. 8:**
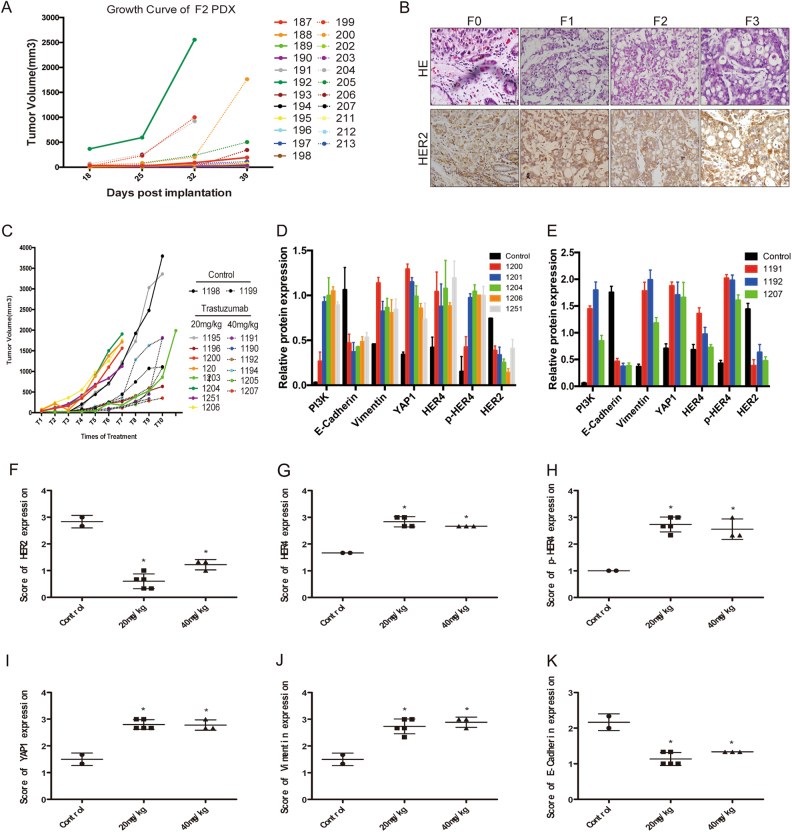
The HER4-YAP1 axis is associated with trastuzumab resistance in a HER2-positive patient-derived xenograft model. **a** The growth curve of F2-generation gastric cancer. **b** HE and HER2 IHC staining from the F0 to F3 generation of the HER2 positive PDX model. **c** The third-generation mice were divided into 3 groups: (1) Control group: received injection of 100 μl PBS via tail injection; (2) 20 mg/kg group: received trastuzumab injection with a dose of 20 mg/kg trastuzumab; (3) 40 mg/kg group: double trastuzumab dosage to the 20 mg/kg. The tumor size was detected every 3 days. **d** The expression levels of HER2, HER4, p-HER4, YAP1, Vimentin, E-Cadherin, and PI3K in selected 20 mg/kg groups were assessed by western blotting. **e** The expression levels of HER2, HER4, p-HER4, YAP1, Vimentin, E-Cadherin, and PI3K in selected 40 mg/kg groups were measured by western blotting. **f**–**k** IHC staining of HER2, HER4, p-HER4, YAP1, Vimentin, E-Cadherin in selected species of control, 20 mg/kg and 40 mg/kg groups, respectively. The data are presented as mean ± SD of three independent experiments

To maximize success rate for development of HER2 targeted therapeutics, PDX model reflecting HER2 positive gastric cancer patients were established. In the general gastric cancer model, we observed that all third-generation tumors began to grow in bursts when post-implantation time reached 28 days (Fig. 8a). HE staining and HER2 IHC staining analyses of the PDX model of HER2-positive gastric cancer showed that histological and pathological features were well-maintained from F0 to F3 generation (Fig. 8b). The F3 generation mice were divided into 3 groups randomly, control group, 20 mg/kg group and 40 mg/kg group. As shown, 3 groups grew quickly and reached to potential clinical resistant endpoint (Fig. 8c). Trastuzumab resistance in the model was confirmed with expression analysis of related proteins (Supplementary Figure 5A). Five candidate trastuzumab-resistant mice were identified in 20 mg/kg dose groups, and 3 were identified in 40 mg/kg dose groups. The western blot analysis revealed decreased expressions of HER2 and E-Cadherin with elevated expressions of p-HER4, YAP1, Vimentin, and PI3K in trastuzumab-resistant mice compare to control group (Fig. 8d, e and Supplementary Figure 5B, C). Consistently, IHC analysis showed the same tendency and confirmed trastuzumab resistance in our PDX model (Fig. 8f–k, Supplementary Figure 6A and Supplementary Table 2). These results indicated that HER4-YAP1 axis acted as a vital pathway that promoted trastuzumab resistance in HER2-positive gastric cancer by inducing the occurrence of EMT.

## Discussion

Ubiquitous resistance developed during drug treatment pose additional challenges to the clinical management of patients with HER2 positive metastatic gastric cancer [9, 10]. However, the underlying mechanism of trastuzumab resistance in HER2-positive gastric cancer remains unclear. By establishing two HER2 positive gastric cancer cell lines that was stably resistant to trastuzumab and by establishing two animal models, we determined that upregulated HER4 proteins interacted with YAP1 to induce EMT could subsequently stimulate cancer cell metastasis. Furthermore, the HER4-YAP1 axis activated the PI3K pathway to maintain tumorigenesis of HER2-positive gastric cancer and escape the block by trastuzumab. To our knowledge, this is the first study to elucidate the mechanism of HER4-YAP1-EMT axis involvement in trastuzumab resistance in HER2 positive gastric cancer.

Recent studies uncovered that EMT indicates drug resistance, namely, trastuzumab resistance, in breast cancer [27–29]. In accordance with the findings of the present study, our established trastuzumab-resistant cell lines were observed obvious acquisition of the mesenchymal morphology, decreased levels of epithelial markers and increased levels of mesenchymal marker Vimentin. Further investigation confirmed the increased capacity of migration and invasion after trastuzumab resistance acquired.

HER4, a member of the ErbB family, activates the MAPK and PI3K/AKT/mTOR pathways by forming a heterodimer with HER2 or by binding to NRG1 [30, 31]. HER4 activation by its ligand (NRG) leads to auto phosphorylation of its tyrosine residues, along with proteolytic cleavage and accumulation of an intracellular 80-kd fragment (4ICD) [32]. In breast cancer, HER4 knockdown has been suggested to induce apoptosis in cells that are resistant to HER2 inhibitors [23]. Furthermore, nuclear localization of HER4 is associated with acquired trastuzumab resistance [22]. The activation/phosphorylation of ErbB4 receptors and the recruitment of signaling effectors are significantly affected by the type of the ligands that bind to the ErbB receptors. It has been shown that the complement of signaling effectors recruited to and activated by ligand-stimulated and phosphorylation HER4 was considered as the really working status [33]. Our study confirmed that HER4 was upregulated and phosphorylated in resistant cells compared to parental cells. More importantly, silencing HER4 inhibited the occurrence of EMT and PI3K signal. Besides, it also increased apoptosis of resistant cells, reduced tumor growth and inhibited metastasis in nude mice, suggesting a key role HER4 played in acquired resistance.

Haskins[34] revealed that Neuregulin-1 (NRG1)-activated HER4 interacts with YAP1 could promote cell migration, connecting the nuclear function of HER4 to the mesenchymal pathway. As a potential target of HER4, YAP1 also promotes EMT and the consequent aggressive phenotype [35]. Our results showed YAP1 level was correlated with the HER4 expression, indicating that HER4 might interact with YAP1. Further co-immunoprecipitation and confocal microscopy confirmed the directly interaction and co-localization existed between HER4 and YAP1. To explore the role of YAP1 in HER4-mediated EMT and trastuzumab resistance, recovery assay was conducted and indicated that HER4 exerts its EMT effects on trastuzumab-resistant cells through regulating its downstream YAP1.

NRG1 is well recognized to induce HER2-HER3 dimer overexpression not only in HER2 positive breast cancer but also in HER2 positive gastric cancer [36]. Recent studies uncovered that HER4 still play a vital role in inducing trastuzumab resistant [24]. In our research, we demonstrated that NRG1 absolutely promoted the expression of HER4 and enhanced the trastuzumab resistance by regulating EMT. With stimulation of NRG1 in a specific time, HER4, p-HER4, YAP1, Vimentin, and PI3K expressions increased and E-Cadherin decreased, indicating the vital role of NRG1 in promoting resistant cells invasion and migration capacity. Furthermore, silencing YAP1 could reverse the function of NRG1 and promote resistant cells apoptosis.

Recently, the PDX model was shown to be highly consistent with the biological and genomic characteristics of patients [37, 38]. In our study, a HER2-positive gastric cancer-derived tumor xenograft model was established. Continual stimulation of trastuzumab was performed to model the drug resistance process in human gastric cancer. It was observed that evident increased of HER4 expression and p-HER4, which was accompanied by increased YAP1 expression, existed in the trastuzumab-resistant model. EMT was also observed in our analysis of tumor samples from the trastuzumab-resistant mice. Due to the absence of an effective HER4-targeted drug, we did not combine HER4 inhibitors with trastuzumab to counteract resistance to the anti-HER2 treatment.

In conclusion, we have put forth the notion that the HER4-YAP1 axis might regulate acquired resistance to trastuzumab in HER2-positive gastric cancer cells by promoting EMT. The axis confers an increased proliferation capacity to HER2-positive gastric cancer cells exposed to trastuzumab. The abnormal expression of HER4 and YAP1 increase activation of downstream signaling pathways PI3K to maintain the aggressive phenotype and promote resistance to the trastuzumab treatment. Our study might elucidate the mechanism of trastuzumab resistance in gastric cancer and provide a promising strategy that would benefit patients with gastric cancer who develop trastuzumab refractory disease.

## Materials and methods

### Cell culture

MKN45/NCI-N87 were purchased from ATCC and maintained as described. The cells were cultured in RPMI 1640 medium (Hyclone) containing 10% fetal bovine serum (FBS, Gibco-BRL; Invitrogen), 8 mg/mL penicillin and 8000 U/mL streptomycin (HyClone) at 37 °C in a humidified incubator with 5% CO_2_ as described [39]. NRG1 was purchased from R&D systems.

### Cell migration assay

Transwell chamber migration assay was measured using a transwell chamber with 8 μm filter inserts (Corning). 5 × 10^4^ cells were mixed with 0.2 ml of serum-free medium and seeded to the upper chambers of transwell plates (Corning) as described [40]. In the lower chamber, 0.6 ml of medium with 10% FBS was added to promote cell movement through the pores of the membrane. The inside of the inserts was cleaned thoroughly with a cotton swab, and cells which had migrated through the porous membrane were fixed with a methanol solution for 15 min, and Giemsa staining was performed. The numbers of cells in 4 randomly selected microscope fields were determined.

### Wound healing assay

A total of 5 × 10^5^ cells were cultured in six-well plates to hungry for 24 h; two perpendicular straight-line scratches were generated on the bottom of the plate. The scratches were photographed every 6 h, and the widths were measured under an inverted microscope.

### Establishment of trastuzumab-resistant cells and a lentivirus-transfected cell line

Increasing doses of trastuzumab were gradually added to the culture medium after 2-day intervals with trastuzumab-free medium until the cell line became stably resistant to all concentrations of trastuzumab, as previously reported [15]. Resistant cells to trastuzumab were maintained with 1 µg/ml trastuzumab. Additionally, a lentivirus system (Genechem, Shanghai) was employed to stably knockdown the HER4 expression in resistant cells, and cells were also transfected with negative-control viruses as the NC. Q-PCR and western blot analyses were performed to detect the HER4 expression after 3 days.

### Western blotting analysis

Protein from cells and tissues was separated by SDS-PAGE and transferred to PVDF membranes as described [41]. The following primary antibodies were used for western blots: the monoclonal rabbit HER4 antibody (1:500, Santa Cruz); the HER2 antibodies and the p-HER4 antibody (1:500, Bioss); the E-cadherin, Vimentin, Cleavage-caspase3, Cleavage-caspase9, YAP1, and GAPDH antibodies (1:500, Proteintech Group); and PI3K antibodies (1:1000, Cell Signaling Technology). The proteins were visualized using the Luminata Chemiluminescent Detection Kit (Millipore).

### RNA isolation and real-time quantitative PCR analysis

Total RNA from tissues or cells was extracted using the Trizol reagent, following by reverse transcription for purified cDNA templates. A SYBR Premix Ex Taq II Kit (Takara) was used to perform real-time Q-PCR in the presence of oligo dT primers (Invitrogen) according to the manufacturer’s recommendation. The sequences of human primers are summarized in Supplementary Table 1. The mRNA expressions were normalized to glyceraldehyde 3-phosphate dehydrogenase (GAPDH).

### Immunohistochemistry and HE analysis

As previously described, immunohistochemistry (IHC) was performed to investigate protein expression in tissue [42, 43]. Tumor samples were obtained from nude mice and NSG mice. The sections of IHC were then incubated with antibodies against HER4, p-HER4, HER2 (1:100), E-cadherin, Vimentin, and YAP1(1:50). Intensity of staining of cancer cells was scored as follows: 0 (no staining), 1 (weakly staining, light yellow), 2 (moderately staining, yellowish brown), and 3 (strongly staining, brown). An intensity score of ≥2 was considered as overexpression, whereas <2 in the intensity score was regarded as low expression. The discrepancies (<5%) were resolved by simultaneous reevaluation. Hematoxylin and eosin (HE) staining was performed to verify the presence of cancer cells, and the results were calculated as the area of the metastatic lesion. All evaluation was analyzed by three independent observers using the same light microscope.

### Co-immunoprecipitation

MKN45TR and NCI-N87TR cell lysates were centrifuged, and each supernatant was added to a 50% protein A/G agarose bead solution at a ratio of 100 μl of the bead solution to 1 ml of the sample solution. When the proteins that non-specifically bound to the beads were cleared, the protein A/G agarose beards were discarded by transferring the supernatant to a separate tube. The HER4 and YAP1 antibodies were used to pull down the proteins that interacted with HER4 and YAP1, respectively, for western blotting; the IgG protein served as the positive control.

### Immunofluorescence assays

Cells were fixed in a 4% paraformaldehyde solution and added a 1% Triton solution to penetrate the cytomembrane. After incubating with antibodies overnight at 4 °C, a fluorescent secondary antibody and the DAPI staining kit were used in the dark to detect primary antibody binding and the cell nuclear, respectively. The inverted microscopy was used to document the expression of the two proteins. After co-incubating cells with the HER4 mouse antibody HER4 (1:50, Novusbio, USA) and YAP1 rabbit antibody YAP1(1:50, Proteintec Group), confocal microscopy was performed to visualize fluorescence.

### Flow cytometry analysis for apoptosis

Cells were seeded to six-well plates at a density of 5 × 10^5^ cells/ml and incubated for 24 h. Then cells precipitates were collected by centrifugation and added binding buffer to resuspend the sediment. Afterward, fluorescein isothiocyanate (FITC) and propidium iodide (PI) were added to the suspension, and cells were incubated per the manufacturer’s suggestion (Annexin V, KIGREN, USA). Flow cytometric analysis (RD) of apoptosis was performed under the guidance of an experienced technician.

### Nude mouse tumor transplantation model

A total of 5 × 10^6^ cells in 0.1 ml of PBS was injected subcutaneously into the right flanks of nude female mice (eight mice per group). Tumors were measured using calipers twice per week; the longest diameter (A) and the shortest diameter (B) of every tumor were recorded to calculate the tumor volumes as follows: *π*/6 × A × B^2^. A total of 5 × 10^5^ cells in 0.1 ml of PBS were injected via the tail veins of mice (eight mice per group) to evaluate metastasis. The mice were killed, and the tumors, livers, and lungs were collected after 1 month. The metastatic lung nodules were observed under the microscope. The magnification areas in the lung was calculated by Image J in three randomly selected microscope fields was determined. All nude mouse experiments were approved by the ethics committee of the Southern Medical University.

### Establishment of the PDX model

PDX model was established to simulate the conditions of gastric cancer evolution in vivo as described [38]. A block of the tissue sample was sectioned into small pieces of ~1 cubic millimeter to be subcutaneously transplanted into the rear leg of NCG mouse, after deep anesthesia was induced by inhalation of isoflurane. A similar operation was performed to transfer the tumor piece to the next passage when the tumor size reached the cutoff value (15×15 mm). Patients with HER2-positive gastric signet-ring cell carcinoma without preoperative chemo-radiotherapy was selected to establish HER2 positive PDX model. Trastuzumab was injected twice per week 28 days after the transplantations until the tumor grew to the cutoff volume for sacrifice. All clinical sample collections were performed after obtaining informed consent from the corresponding patients and were approved by the ethics committees of NanFang Hospital (Guangzhou, China). The animal experiments were performed in accordance with the National Institutes of Health Guide for the Care and Use of Laboratory Animals with the approval of the Guangdong laboratory animal monitoring institute.

### Statistical analysis

Data were analyzed using the Student’s* t*-test or one-way analysis of variance, followed by the Student–Newman–Keuls test using SPSS v20.0 statistical software (SPSS, Inc. Chicago, IL, USA) and the results are expressed as the mean ± standard deviation (SD). A two-tailed probability (p)-value of <0.05 was considered statistically significant.

## Electronic supplementary material

Supplementary Figure 1(TIF 5610 kb)

Supplementary Figure 2(TIF 6975 kb)

Supplementary Figure 3(TIF 6939 kb)

Supplementary Figure 4(TIF 2734 kb)

Supplementary Figure 5(TIF 5144 kb)

Supplementary Figure 6(TIF 12975 kb)

Supplementary Table(DOCX 289 kb)
