# Supplementary figures and images for: The HER4-YAP1 axis promotes trastuzumab resistance in HER2-positive gastric cancer by inducing epithelial and mesenchymal transition
Source: Oncogene. 2018 Mar 14;37(22):3022–38. doi: 10.1038/s41388-018-0204-5 (PMC5978807; doi:10.1038/s41388-018-0204-5)

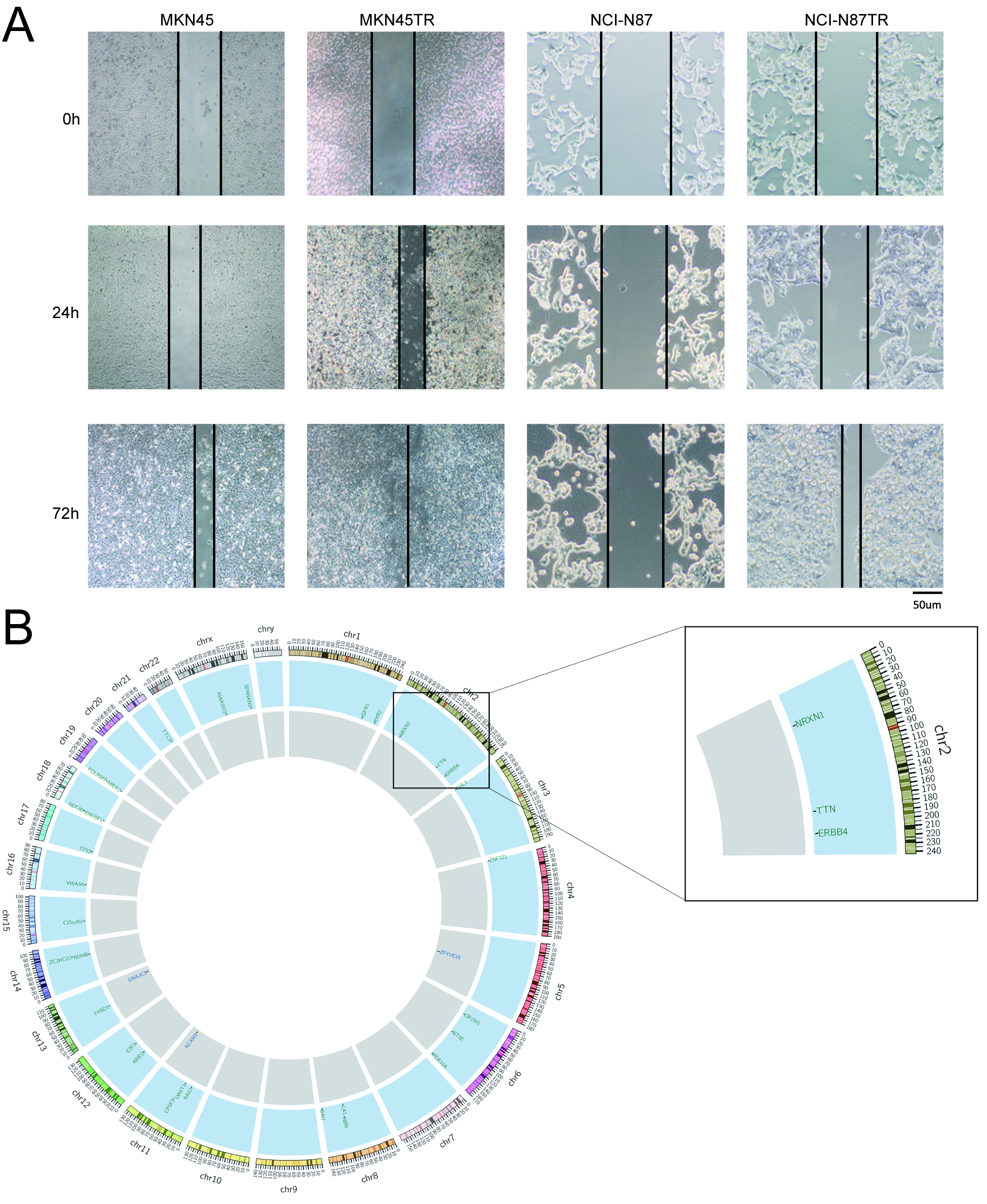

Supplement: Supplementary file 1 — Supplementary Figure 1(TIF 5610 kb) [file 41388_2018_204_MOESM1_ESM.tif]

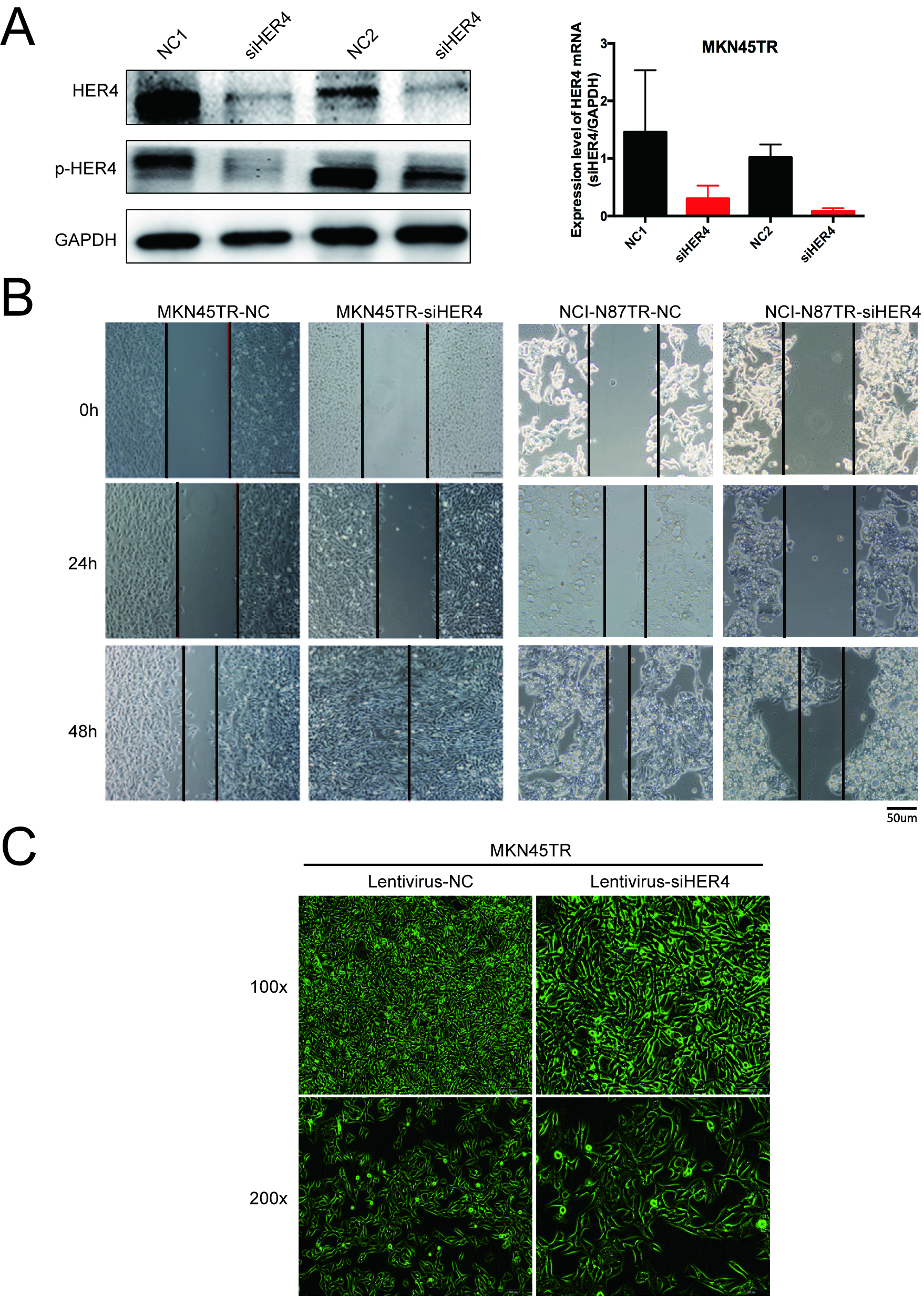

Supplement: Supplementary file 2 — Supplementary Figure 2(TIF 6975 kb) [file 41388_2018_204_MOESM2_ESM.tif]

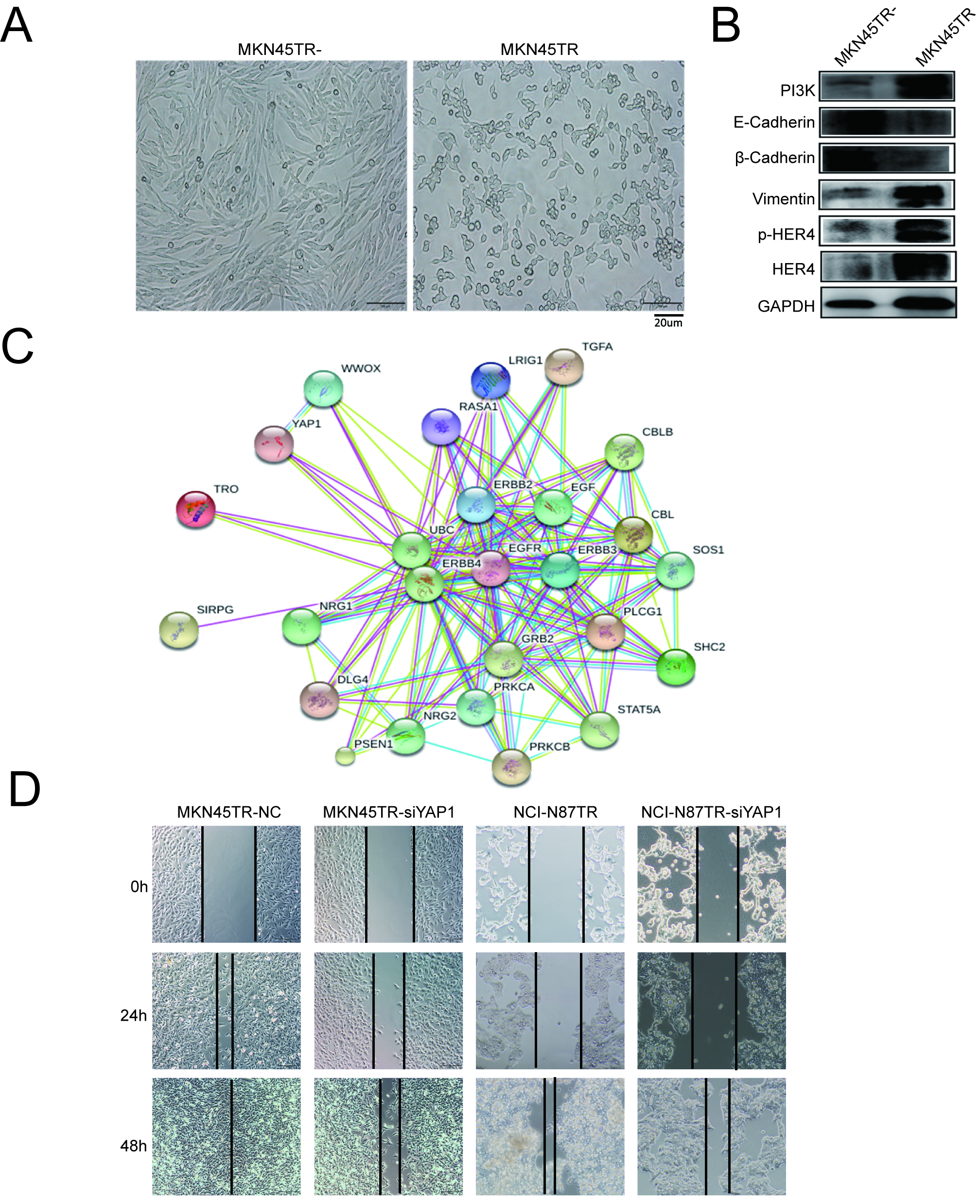

Supplement: Supplementary file 3 — Supplementary Figure 3(TIF 6939 kb) [file 41388_2018_204_MOESM3_ESM.tif]

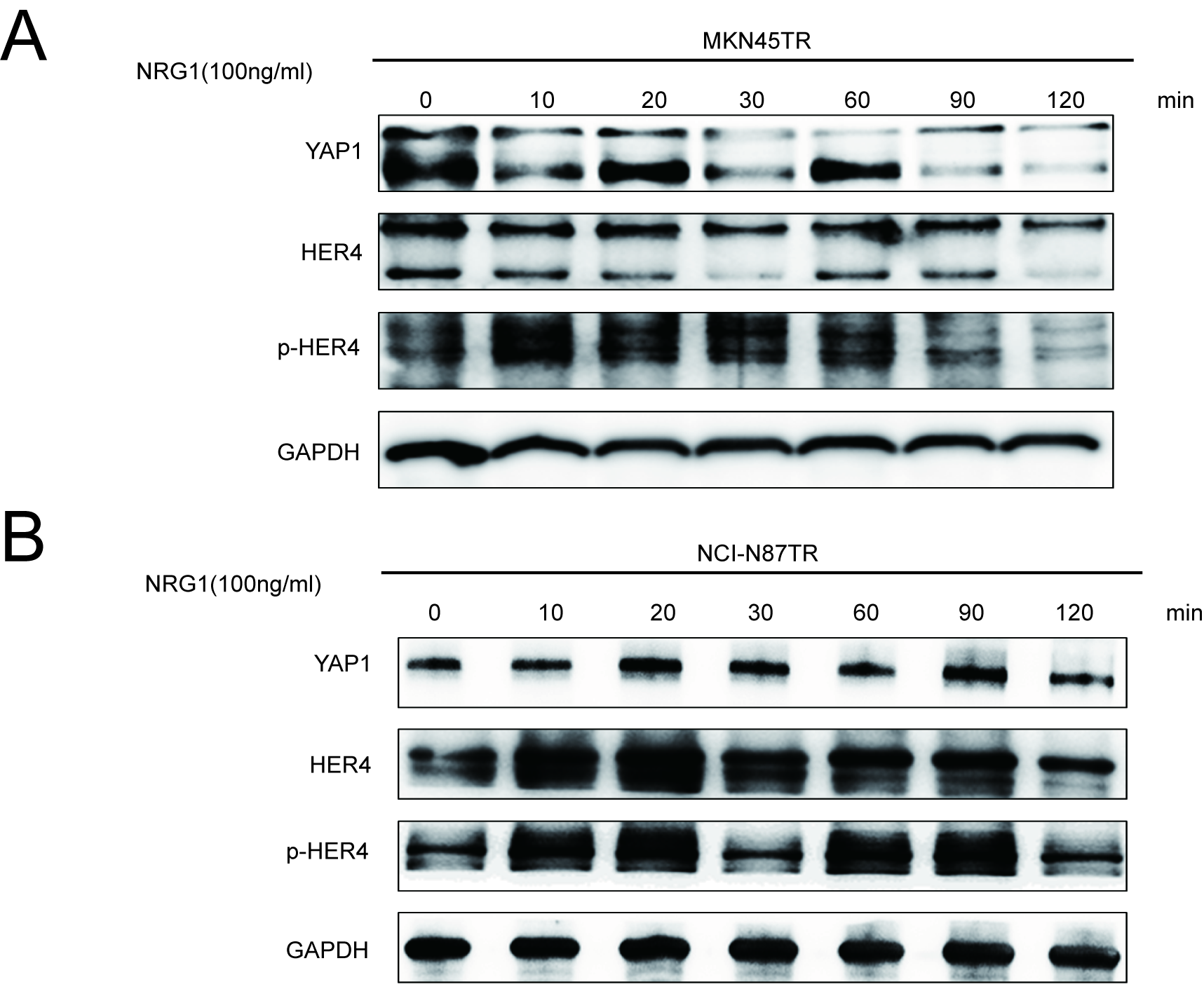

Supplement: Supplementary file 4 — Supplementary Figure 4(TIF 2734 kb) [file 41388_2018_204_MOESM4_ESM.tif]

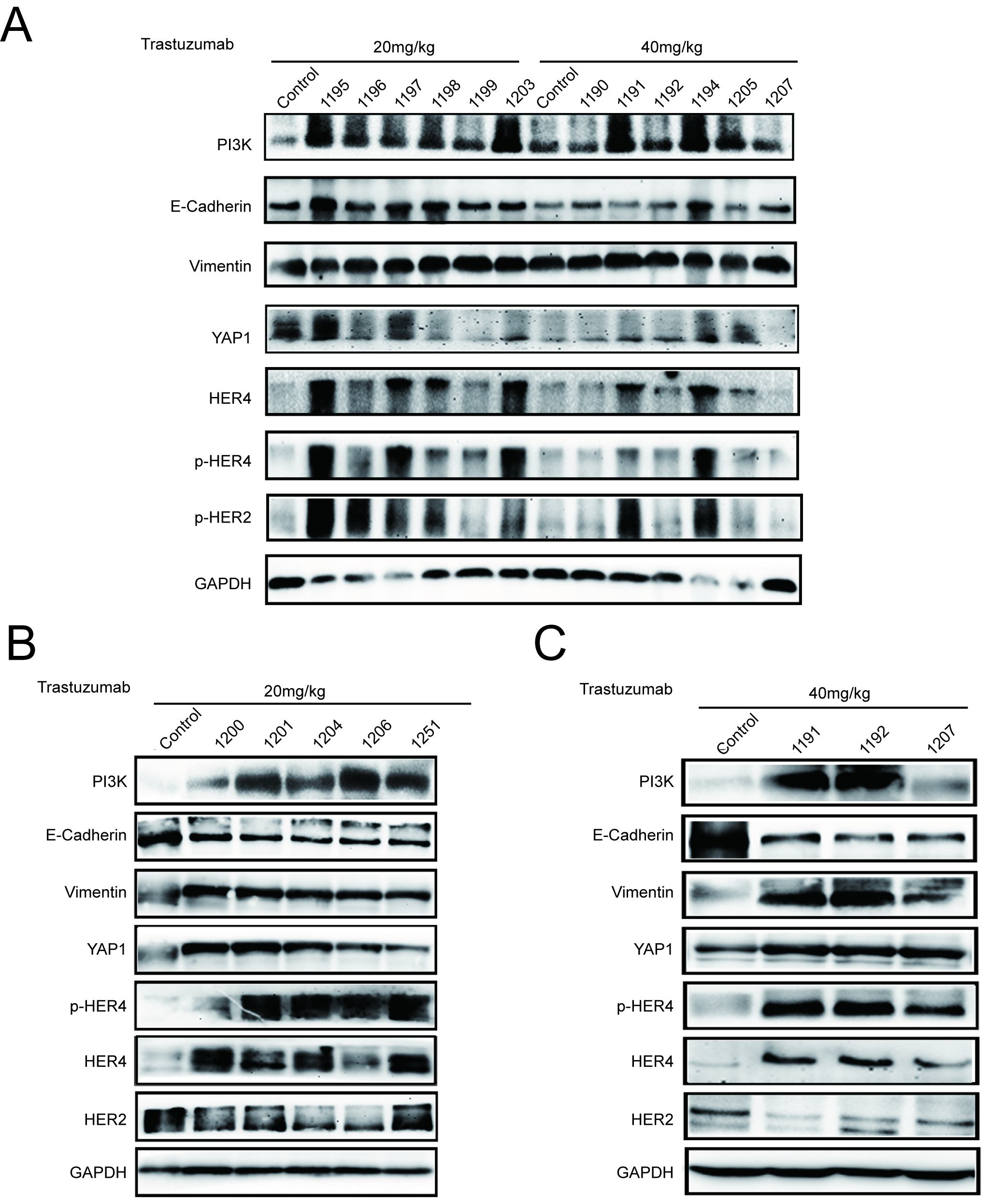

Supplement: Supplementary file 5 — Supplementary Figure 5(TIF 5144 kb) [file 41388_2018_204_MOESM5_ESM.tif]

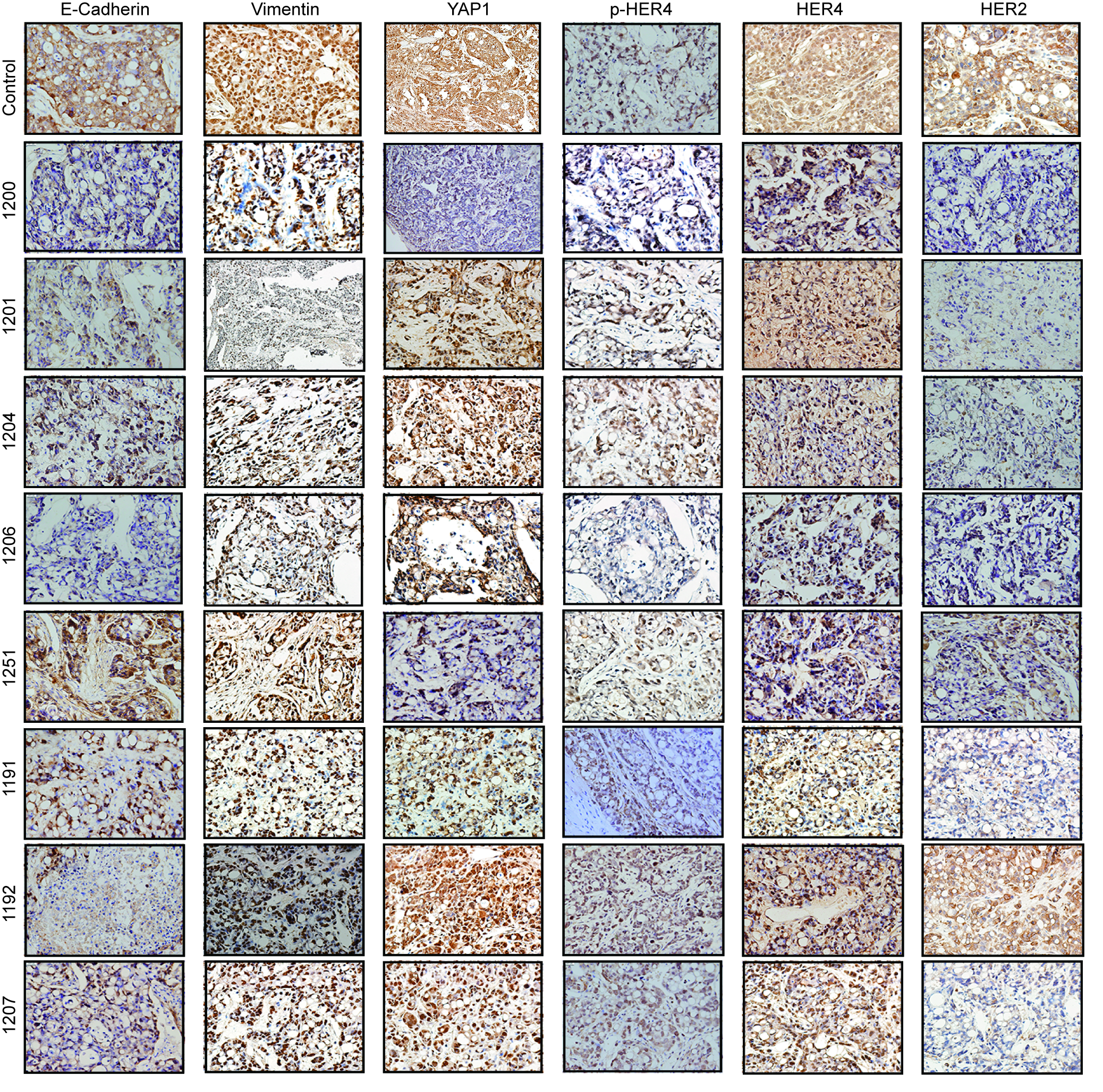

Supplement: Supplementary file 6 — Supplementary Figure 6(TIF 12975 kb) [file 41388_2018_204_MOESM6_ESM.tif]
