# Supplementary material for: The HER4-YAP1 axis promotes trastuzumab resistance in HER2-positive gastric cancer by inducing epithelial and mesenchymal transition
Source: Oncogene. 2018 Mar 14;37(22):3022–38. doi: 10.1038/s41388-018-0204-5 (PMC5978807; doi:10.1038/s41388-018-0204-5)
Supplement: Supplementary file 7 — Supplementary Table(DOCX 289 kb) [file 41388_2018_204_MOESM7_ESM.docx]

Supplementary Table 1: siRNA and primer sequences

Supplementary Table 2: IHC score for potential resistant PDX model in Excel file
